# A Feasibility Study of Transformer Winding Temperature and Strain Detection Based on Distributed Optical Fibre Sensors

**DOI:** 10.3390/s18113932

**Published:** 2018-11-14

**Authors:** Yunpeng Liu, Yuan Tian, Xiaozhou Fan, Yanan Bu, Peng He, Huan Li, Junyi Yin, Xiaojiang Zheng

**Affiliations:** 1 State Key Laboratory of Alternate Electrical Power System with Renewable Energy Sources, North China Electric Power University, Baoding 071003, China; liuyunpeng@ncepu.edu.cn; 2 Hebei Provincial Key Laboratory of Power Transmission Equipment Security Defence, North China Electric Power University, Baoding 071003, China; tianyuan_274@126.com (Y.T.); buyn94@sina.cn (Y.B.); 15243463653@163.com (P.H.); Xenon_Li@126.com (H.L.); jy_yin@126.com (J.Y.); 3 State Grid Hebei Electric Power Company, Shijiazhuang 050021, China; zhengxj@he.sgcc.com.cn

**Keywords:** transformer winding, temperature, strain, distributed optical fibre, Brillouin scattering, Raman scattering

## Abstract

The temperature distribution and deformation of the transformer windings cannot be measured in a distributed manner by the traditional method and failure location cannot be performed. To solve these problems, we present a transformer winding temperature and strain based on a distributed optical fibre sensing detection method. The design of the optical fibre winding composite model is developed and simulated winding temperature rise test and local deformation test distinguish between measuring the winding temperature and the strain curve. The test results show that the distributed optical fibre can transmit wire strain efficiently. Optical fibres, in the process of winding, have a certain pre-stress. Using the Brillouin–Raman joint measuring method, one can effectively extract the optical fibre temperature and strain information and measure the length of the winding direction of the temperature and strain distribution curve to a temperature measurement precision of ±2 °C and strain detection accuracy of ±50 με. The system can carry out local hot spot and deformation localisation, providing new ideas for the transformer winding state monitoring technology.

## 1. Introduction

Power transformers play an important role in power systems and their safe operation has a direct influence on the reliability and safety of the power supply. Statistics show that insulation damage is the main cause of transformer failure with the winding being the part with the highest failure rate. The winding temperature and accurate and real-time deformation checks are significant to the safe operation of transformers.

Currently, there are several ways to check transformer temperature such as the top oil temperature measuring methods [1,2,3], fluorescence optical fibre temperature measuring methods [4,5], fibre Bragg grating methods, etc. [6,7,8,9]. The measuring scope of the top oil temperature measuring method is small due to its low measurement accuracy; fluorescence optical fibre temperature measuring offers a higher measuring accuracy, but it is a point-type measure and is still limited in scope since the number of sensors has to be increased to measure different parts; the fibre Bragg grating method is a point-type measure though, in essence, it can make quasi-distributed measurements. The one optical fibre has a limited number of gratings, so it is difficult to make long-distance measurements.

The main methods to diagnose winding deformation off-line are the short-circuit impedance method [10], low-voltage pulse method, and frequency response analysis method [11]: however, it is difficult for off-line detection to meet the development trend of on-line detection and state evaluation of electric equipment; besides, it suffers from poor sensitivity and difficulty in recognising winding deformation. In addition, the live detection method for transformer winding using bushing tap injection is still under investigation [12,13]. There are some problems such as the isolation between the external system response and the transfer function of the winding, and the impact of load variation on the measurement results.

With advantages such as a distributed measuring method, long measuring distance, resistance to electromagnetic interference, and high insulation strength [14], distributed optical fibre sensing technology is widely applied to the state measuring of large matrices in architecture [15], bridges, and slopes. In electrical engineering, it is also applied to measuring the temperature and strain of electric equipment. References [16,17,18] present the structure of optical fibre composite power cable and introduce the application of distributed optical fibre temperature sensing technology in cable temperature detection. The integration of optical fibres to power cables is implemented as being located in special non-magnetic tubes that are placed between screening wires of high voltage cables. The technology to build fibre into power cables is ripe. However, compared with the cable, the transformer size and the deformation degree is much smaller. The difficulty lies in the fibre laying method, which should not influence the normal operation of the transformer, and ensure that the fibre and the conductor are at the same temperature and at a synchronous deformation.

At present, fibre sensing technology has been applied in transformer detection, for example, fluorescent fibre measurements and quasi-distributed fibre temperature measurements using fibre Bragg grating (FBG) [19,20,21,22]. Reference [20] presents a method of combining fibre with winding wire. However, the above methods are all point temperature measurement, so the temperature distribution of transformer windings cannot be mastered comprehensively. There is no report on the inspection of temperature fields and strain in transformer windings based on the distributed optical fibre sensing technology.

In this paper, the distributed optical fibre inspection method for the temperature and strain of transformer winding based on Brillouin–Raman joint measuring is presented, and an optical fibre composite wire is designed and made to realise the simultaneous measurement of winding temperature and strain, obtain a distribution curve of winding temperature and strain, and locate local hot-spots and deformations so as to provide new research ideas for the development of monitoring technology as applied to transformer states.

## 2. Detection Principle

**Figure 1:**
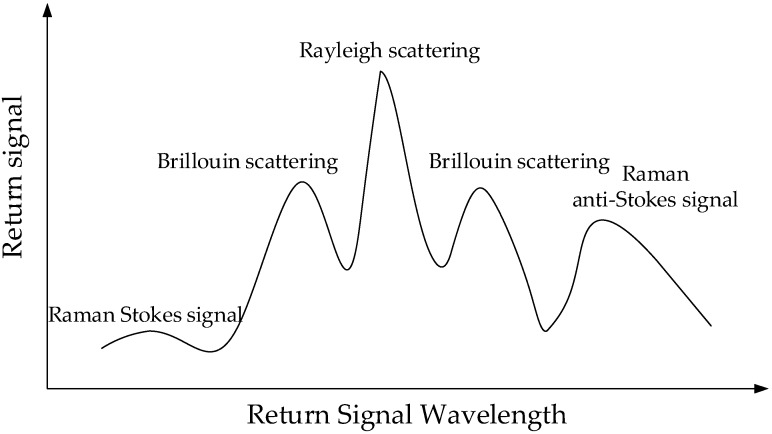
The scattering in the optical fibre.

The incident pulse light will experience Rayleigh scattering, Brillouin scattering, and Raman scattering, as shown in Figure 1. Raman scattering is only sensitive to temperature and it is divided into Stokes and anti-Stokes scattered light. Anti-Stokes scattered light is sensitive to temperature while Stokes light is less affected by temperature, and the intensity of these two kinds of scattered light is in direct proportion to the temperature change [23]:

(1) $$\frac{I_{\mathrm{as}}}{I_{s}}={\left(\frac{\lambda_{s}}{\lambda_{as}}\right)}^{4}\exp\left(-\frac{B}{T}\right)\;$$

where

(2) $$B=\frac{hc\upsilon }{k}\;$$

where *I*_as_ is the anti-Stokes light intensity; *I*_s_ is Stokes light intensity; Parameters λ_s_ and λ_as_ are Stokes and anti-Stokes optical wavelengths, respectively; *c* is the velocity of light in vacuo; *h* is the Planck coefficient; *T* is the temperature; *k* is the Boltzmann constant, and *v* is the Raman offset.

The temperature at a measuring point can be obtained by measuring and calculating the intensity ratio between Stokes and anti-Stokes light.

The frequency shift of Brillouin scattering is related to the speed of sound in the optical fibre. The sound velocity can be affected by the thermo-optic effect and the elasto-optical effect which are related to refraction rate, Young’s modulus, Poisson’s ratio, and the density of the optical fibre material, so the temperature and strain in the optical fibre can both lead to changes of the Brillouin frequency shift and intensity. Its result is represented by the good linearity between the axial strain and temperature and the Brillouin frequency shift of the optical fibre, i.e.,

(3) $$v_{B}\left(T,\varepsilon \right)=v_{B0}\left(T_{0},\varepsilon_{0}\right)+C_{vT}\Delta T+C_{v\varepsilon }\Delta \varepsilon \;$$

where, *υ_B_* (*T*,*ε*) is the Brillouin frequency shift of the optical fibre with temperature *T* and strain *ε*; *υ_B0_* (*T*_0_,*ε*_0_) is the Brillouin frequency shift of the optical fibre under initial temperature *T*_0_ and initial strain ε_0_; *C_υT_* and *C_υε_* are the temperature and strain response coefficients of the Brillouin frequency shift; Δ*Τ* and Δ*ε* are the changes relative to the initial temperature and initial strain.

When the temperature and strain of the optical fibre are measured with Brillouin scattering, an effective distinction between the temperature and strain sensing information must be made. Here, a Brillouin–Raman joint measuring method is adopted, the strain sensing optical fibre and temperature sensing optical fibre of the same length are laid in the same thermal environment, and accurate temperature Δ*Τ* and strain Δ*ε* are obtained by solving Formulas (1)–(3):

(4) $$\left\{ \begin{array}{l} \Delta T=T_{R}-T_{R0}\\ \Delta \varepsilon =\frac{v_{B}\left(T_{R},\varepsilon \right)-v_{B0}\left(T_{R0},\varepsilon_{0}\right)-C_{vT}\Delta T}{C_{v\varepsilon }} \end{array}\right.\;$$

where *T_R_* and *T_R_*_0_ are the target temperature and the initial temperature obtained by Raman scattering measurements, respectively.

## 3. Stability Analysis of Distributed Optical Fibres within the Transformer

### 3.1. The Design of Optical Fibre Composite Wire

**Figure 2:**
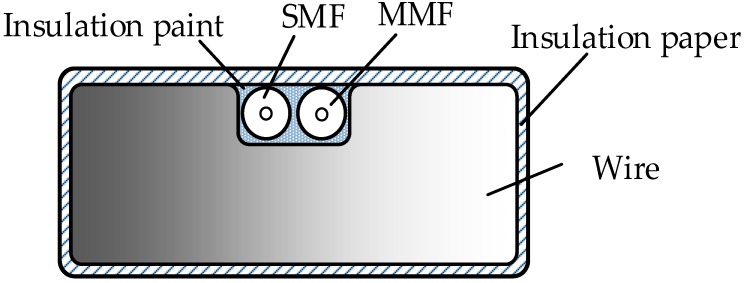
The cross-section of fibre composite wire.

One kind of optical fibre composite wire structure is designed in this paper. A groove is cut in the centre of the wide edge of the wire. A single-mode fibre (SMF) and a multi-mode fibre (MMF) are put on the groove (Figure 2). The SMF is used as the strain-sensing optical fibre and the MMF is used as the temperature-sensing optical fibre. The fibres are fixed in the groove by insulating paint cured to ensure that the optical fibre and wire are deformed synchronously and at the same temperature. Since the optical fibre diameter is small and the area of the wire grooving is less than 2% of the cross-sectional area, the current carrying capacity and mechanical strength of the wire is negligible. Apart from the winding inside the transformer, there are mechanical parts such as the iron core, yoke, and clamping piece as well as interference factors such as transformer oil flow and machine vibration. Traditional electric measurements are usually affected by those factors mentioned above, thus the lower measuring accuracy; however, the inspection frequency of optical fibre measurements is usually above 10 GHz and is unaffected by the machine vibration signal. Meanwhile, the strain inspection optical fibre is integrated with the wire and is deformed synchronously with the wire, and the measured strain curve is only related to the deformation of the wire itself. To ensure the stable working of the optical fibre in high temperatures in the transformer, a high-temperature resistant optical fibre coated with a polyimide is used as it is stable at *T* > 200 °C.

### 3.2. The Simulation of Inter-Turn Electric Field

**Figure 3:**
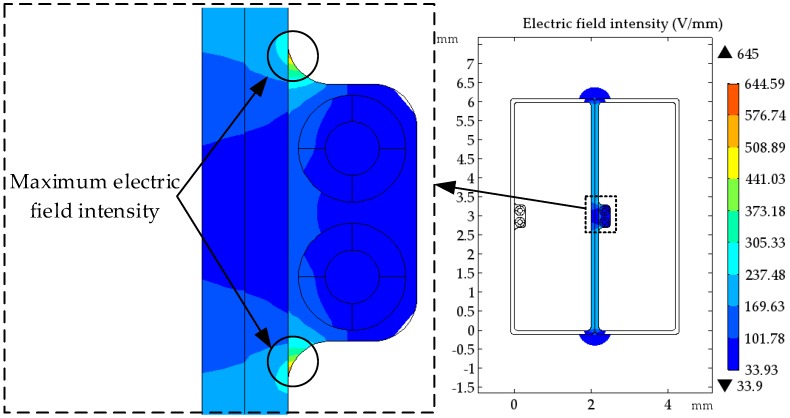
The inter-turn electric field of optical fibre composite wire.

Since winding deformation mostly occurs in low-voltage windings, the wire used in the low-voltage winding of an SFSZ7-31,500/110 kV transformer is modelled to establish a 2-d groove wire model. The voltage grade of the low-voltage winding is 10.5 kV. The wire is 2 mm wide, 6 mm high, the groove is 0.3 mm both in width and depth, and the wide surface is covered in a 0.2-mm thickness of insulation paper. The optical fibre has a double-layer structure with 0.125 mm of fibre core diameter and 0.25 mm of coating layer diameter. As for the 10.5 kV low-voltage winding, the potential difference across two adjacent turns is about 40 V. The relative dielectric constant of each material is listed in Table 1. The inter-turn electric field of the wire after optical fibre installation is shown in Figure 3. It is shown that the maximum electric field intensity after the wire grooving is located at the round corner of the groove (644.59 V/mm), which is 119.6% higher than the inter-turn electric field intensity. The average value of the electric field intensity in the groove is 40% that of the inter-turn electric field intensity. It is far from being enough to affect the insulation performance of the oil paper.

### 3.3. The Power Frequency Resistance Test of the Groove Wire

**Figure 4:**
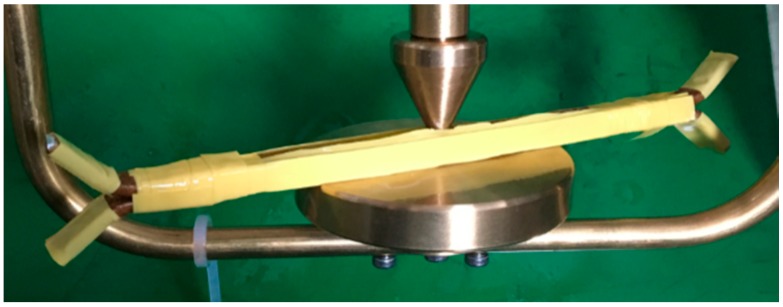
The frequency breakdown voltage test model.

To check the influence of actual wire grooving on insulation, an inter-turn power frequency voltage breakdown test is conducted on the wire before and after grooving. Three layers of insulation paper are added between the wires (Figure 4). The power frequency voltage breakdown test is conducted on the wire before and after grooving and the mean value of ten test results is taken. The test shows that the mean values of power frequency breakdown voltage before and after wire grooving are 6.82 kV and 6.75 kV, respectively, the breakdown positions are all at the edge of the wire and there is no breakdown at the groove. Therefore, it can be considered that the grooving of the wide side of the wire has no influence on winding insulation performance.

## 4. Optical Fibre Strain Transfer

### 4.1. The theoretical Calculation of Embedded Optical Fibre Strain Transfer

Since the current direction in high- and low-voltage windings is opposite when the transfer is subject to a short-circuit electromotive force, the direction of action of the axial short-circuit force between two windings mutually repels it [24]. The low-voltage winding is subject to an inward compressive stress around its whole circumference. Since the winding often is wound on the stay, the wire between two adjacent stays will also produce bending stress under the action of axial short-circuit force. When the fibre is deformed synchronously with the wire, the fibre will be subjected to the combined action of compressive and bending stress.

There has been some research into the strain transfer calculation produced by the uniform axial force applied to the embedded fibre and matrix [25]. When the distance from the end is more than 0.0125 m, the optical fibre strain transfer coefficient is 1. According to the theory of material mechanics, since the diameter of the transformer winding is much greater than the wire width and thickness, the wire can be taken as a small curvature beam to calculate the normal stress in bending [26].

Now the bending strain transfer coefficient is calculated and the following assumptions are made:

1. Each interface of the sensor is always tightly connected under bending.
2. Materials of different layers are all isotropic, linear elastic, bodies.
3. The optical fibre centroid coincides with that of the glue layer.
4. Both the wire and groove have no rounded corners.

**Figure 5:**
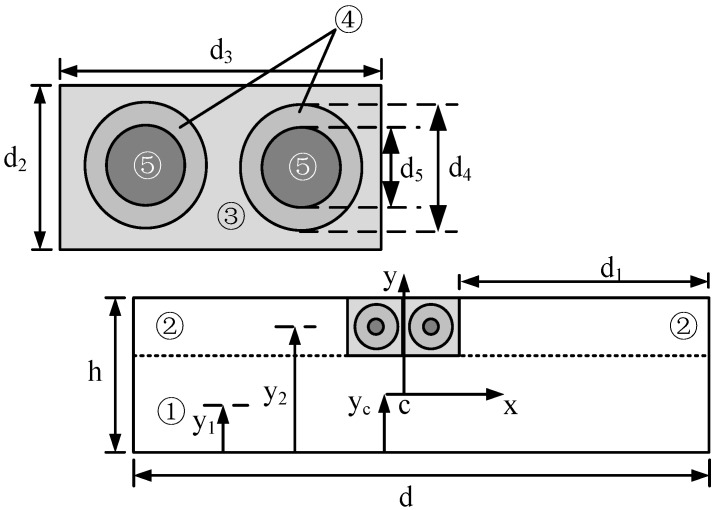
The cross-section of the fibre composite wire.

When the wire is subject to axial short-circuit action, the parameters of the embedded optical fibre wire are shown in Figure 5. The intersection of the axis of symmetry and the neutral axis layer of the model section is taken as the origin of the coordinates. To simplify the calculation, the wire section is divided into five areas and *y*_1_, *y*_2_, *y*_3_, *y*_4_, and *y*_5_ are the distances from the centroid of each part to the wire bottom, thus *y*_2_ = *y*_3_ = *y*_4_ = *y*_5_.

Accordingly:

(5) $$\sigma_{i}=\frac{E_{i}y}{\rho }\;$$

where, *σ_i_* (*i* = 1, 2, …, 5) is the stress in the corresponding number of area; *E_i_* (*i* = 1, 2, …, 5) is the elastic module of the corresponding area, and, obviously *E*_1_ = *E*_2_ = *E*_5_; *ρ* is the radius of curvature of the optical fibre composite wire; *y* is the distance between any layer and the neutral axis layer.

In combination with the definition of the static bending moment, the distance *y*_c_ from the neutral layer of the optical fibre composite fibre to the bottom is

(6) $$y_{c}=\frac{\sum_{i=1}^{5}E_{i}A_{i}y_{i}+E_{2}A_{2}y_{2}+\sum_{i=4}^{5}E_{i}A_{i}y_{i}}{\sum_{i=1}^{5}E_{i}A_{i}+E_{2}A_{2}+\sum_{i=4}^{5}E_{i}A_{i}}\;$$

Accordingly, the strain in any layer of the model is

(7) $$\varepsilon =\frac{y}{\rho }=\frac{yM}{\sum_{i=1}^{5}E_{i}I_{i}+E_{2}I_{2}+\sum_{i=4}^{5}E_{i}I_{i}}\;$$

where, *I*_i_ (*i* = 1, 2, …, 5) is the second moment of area about the *x*-axis of each area:

$$\left\{ \begin{array}{l} I_{1}=\frac{d{\left(h-d_{2}\right)}^{3}}{12}+d(h-d_{2}){\left(y_{1}-y_{c}\right)}^{2}\\ I_{2}=\frac{d_{1}d_{2}^{3}}{12}+d_{1}d_{2}{\left(y_{2}-y_{c}\right)}^{2}\\ I_{3}=\frac{d_{3}d_{2}^{3}}{12}+d_{3}d_{2}{\left(y_{3}-y_{c}\right)}^{2}-2(I_{4}+I_{5})\\ I_{4}=\frac{\pi d_{4}^{4}}{64}+\frac{\pi d_{4}^{2}}{4}{\left(y_{4}-y_{c}\right)}^{2}-I_{5}\\ I_{5}=\frac{\pi d_{5}^{4}}{64}+\frac{\pi d_{5}^{2}}{4}{\left(y_{5}-y_{c}\right)}^{2} \end{array}\right.\;$$

According to Equations (6) and (7), the strain at the optical fibre centre is as follows:

(8) $$\varepsilon_{f}=\frac{(y_{5}-y_{c})M}{\sum_{i=1}^{5}E_{i}I_{i}+E_{2}I_{2}+\sum_{i=4}^{5}E_{i}I_{i}}\;$$

The strain at the wire surface is

(9) $$\varepsilon_{m}=\frac{(y_{m}-y_{c})M}{\sum_{i=1}^{5}E_{i}I_{i}+E_{2}I_{2}+\sum_{i=4}^{5}E_{i}I_{i}}\;$$

where, *y*_m_ is the distance from the wire surface to the bottom.

As indicated, the optical fibre strain is related to the distance from the optical fibre centroid to the neutral layer. When the optical fibre is closer to the wire surface, the strain increases and the detection sensitivity to wire deformation is higher. Since BOTDR has a certain spatial resolution, the strain measured in the length of the spatial resolution is the average strain along the gauge length and, therefore, the average value of the strain transfer rate in the perceived length is given by

(10) $$\alpha =\frac{\int_{-L}^{L}\varepsilon_{f}(x)dx}{\int_{-L}^{L}\varepsilon_{m}(x)dx}\;$$

### 4.2. Strain Transfer Test

**Figure 6:**
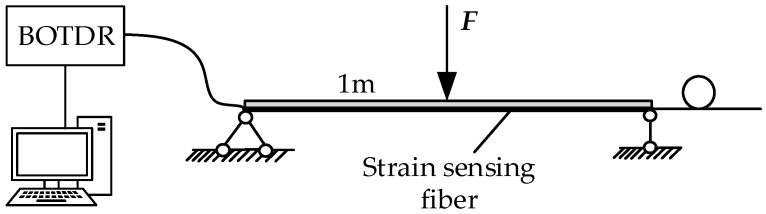
The strain transfer test system.

**Figure 7:**
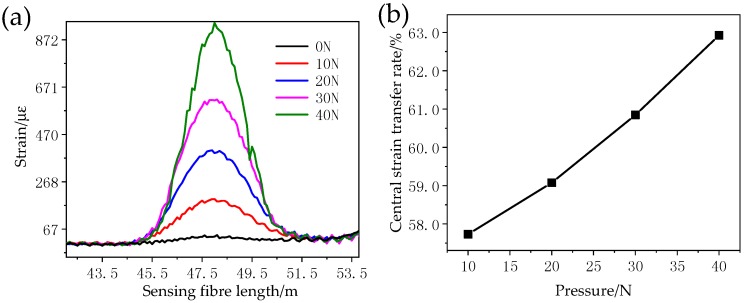
The fibre strain curve (**a**) and central strain transfer rate (**b**) under different pressures.

A groove with a 0.3 mm side-length is cut on the wide side of a straight copper bar which is 1 m long, 30 mm wide, and 3 mm thick according to the method above, and insulating paint is applied to fix the distributed optical fibre in the groove. Both ends of the copper bar are fixed. A vertical downwards force is applied at the centre in 10 N increments over four steps each with an accuracy of 0.01 N (Figure 6). The Brillouin optical time domain reflectometer (BOTDR) system is used to measure the strain at a constant room temperature. The measurement spatial resolution is 2 m, and the sampling resolution is 0.2 m. Finally, the strain value corresponding to the wire surface at the maximum strain of the optical fibre is taken to calculate the strain transfer coefficient of the fibre at different stresses (Figure 7). The range of the optical fibre strain sensor is set to be 48~49 m.

As shown in Figure 7, since a BOTDR system is limited by its spatial resolution, the strain measured by the optical fibre is actually the comprehensive strain within the spatial resolution length with the measuring point as the start point [27]. The strain on the wire surface can be transferred to the fibre when the test fibre is in synchronous deformation with the wire. The strain curve detected by the optical fibre can reflect the strain state of the winding. The strain variation region is 45~51 m, the pulse width of BOTDR is 20 ns, and the spatial resolution is 2 m, so there is a strain transition distance of approximately 2 m at both ends of the 1 m sensing fibre. Meanwhile, when the measured strain in the optical fibre is smaller than the actual strain, the strain transfer rate increases with increasing stress and it is asymptotic to its theoretical value.

## 5. Detection of the Temperature of, and Strain in, the Transformer Winding

### 5.1. Building the Test Platform

**Figure 8:**
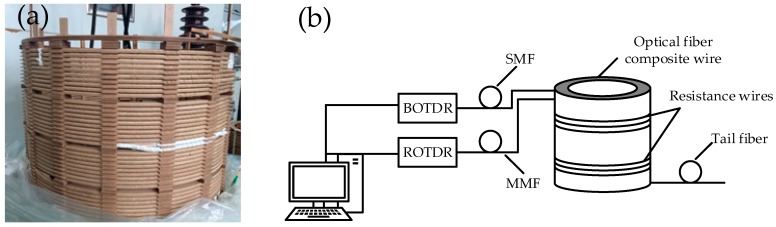
(**a**) The optical fibre winding composite model and (**b**) measurement system.

A spiral winding model is made according to the size of a low-voltage winding of SFSZ7-31,500/110 kV transformer. For convenient deformation setting, eight pieces of wires are wound in parallel and the outermost wire is changed to an optical fibre composite wire made according to the above method. Finally, a winding model with an outer diameter of 700 mm, composed of 40 cakes with a total length of about 90 m is made (Figure 8a). To eliminate the measurement error caused by the head-end blind area and tail-end reflection, a 20 m optical fibre pigtail is connected to the head- and tail-ends of the model. To simulate the uneven distribution of the winding temperature and local overheating in a real transformer, one resistance wire is parallel wound and attached to the outermost wires of the No. 10–12 and 30–32 cakes, over about 20 m in total length. A thermocouple is used to measure the wire surface temperature for comparative measurement (Figure 8b).

BOTDR technology uses a single mode fibre as the sensing element. Due to differences in the optical fibre material and process, the performance parameters of the single-mode tightly buffered optical fibres from different manufacturers, modes, and sheathing materials are different. Therefore, temperature calibration and strain calibration tests have to be conducted. Multiple calibration tests have been conducted for the single mode optical fibre used here: the temperature and strain coefficients obtained are 1.32 MHz/°C and 0.0528 MHz/με respectively.

**Figure 9:**
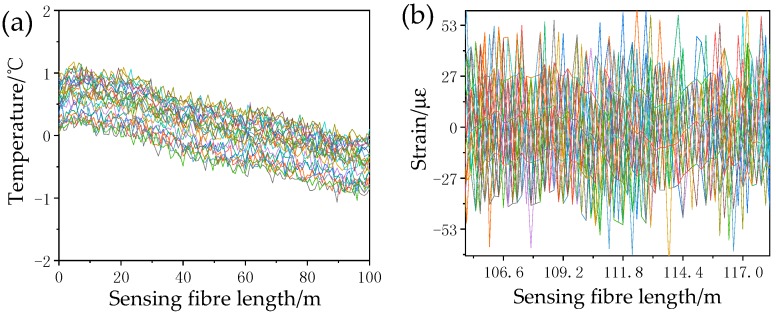
(**a**) The temperature measurement error of the Raman optical time-domain reflectometry (ROTDR) system and (**b**) strain measurement error of the Brillouin optical time domain reflectometer (BOTDR) system.

**Figure 10:**
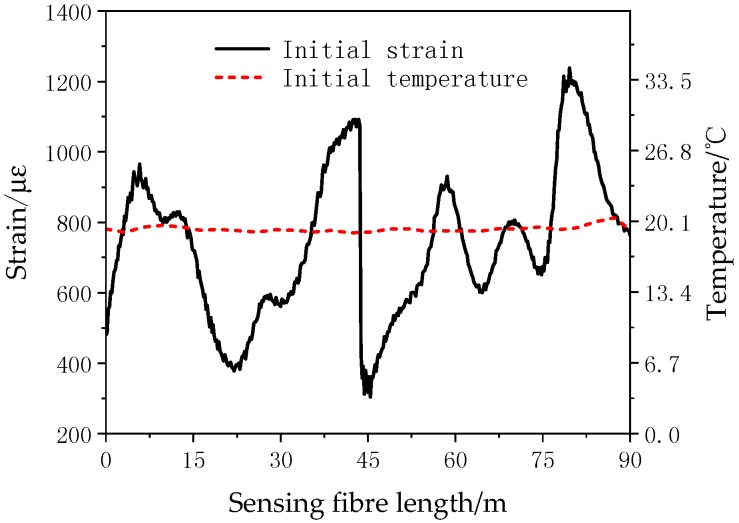
The original strain and temperature of the optical fibre at room temperature.

When making coils, the wire and optical fibre will be influenced by positional changes and pulling. In order to ensure that the optical fibre is not damaged during winding, BOTDR is used to monitor the optical fibre strain. A Raman optical time-domain reflectometry (ROTDR) is used to measure the temperature curve on the winding wire and temperature compensation applied according to Equation (4). BOTDR and ROTDR are produced by WEIHAI BEIYANG OPTOELECTRONIC INFO-TECH CO.LTD. The settings of the instrument parameters are shown in Table 2. The temperature sensing length of the optical fibre is set as 100 m, and the ROTDR system measurement error is ±1 °C in the range of 20–90 °C, as shown in Figure 9a. The optical fibre strain sensor range is set to be 105~120 m, and the measurement error of BOTDR is ±50 με in the range of 0~5000 με as shown in Figure 9b. Figure 10 shows that during the winding of the coil, the strain change is smaller than 1400 με. It is far smaller than the range of optical fibre strain measurement. It suggests that the distributed optical fibre sensor maintained its good strain monitoring performance.

### 5.2. Temperature Rise Test

**Figure 11:**
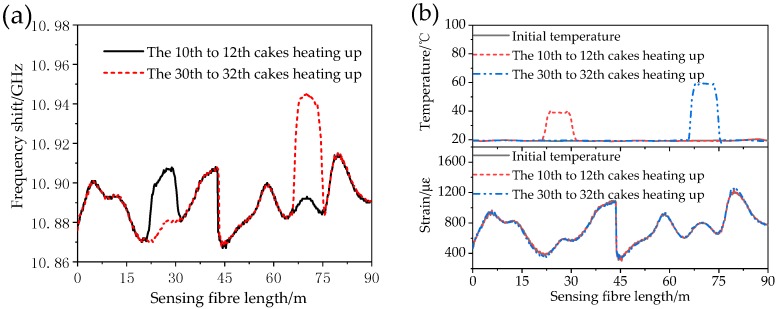
(**a**) The frequency shift curve of BOTDR and (**b**) the temperature and strain curves.

The voltage of the resistance wires on wires of the No. 10–12 cakes and the No. 30–32 cakes increased with a voltage regulator so as to increase the winding temperature to 40 °C and 60 °C, and the temperature of the corresponding wire is measured by thermocouple as a reference. The Brillouin frequency shift curve as measured by the BOTDR system after the winding temperature increase is shown in Figure 11a. Figure 11b shows the optical fibre temperature and strain curve after the temperature compensation with the Raman temperature measuring technology.

To analyse the measurement results, the actual winding temperature rise and actual measurement position, the mean temperature measured by distributed optical fibres for the temperature rise, and the mean temperature measured by the standard thermocouple are compared (Table 3).

The measured position of the temperature change is slightly higher than the actual position because the spatial resolution of the ROTDR system is 2 m and the measured data are the mean temperatures within that spatial resolution. Therefore, there is a temperature response transition distance of 2 m at the sudden temperature change position. The discrepancy between the measured temperature where the winding temperature rises based on the distributed optical fibre sensing and the measured value of the standard thermocouple is <±2 °C. The system is able to locate the point where the winding temperature changes. Meanwhile, the system response time is about 2 to 10 s and it has a fast response to winding temperature change and can reflect the real-time winding temperature distribution.

As shown in Figure 11, the frequency shift curve as measured by the BOTDR system before temperature compensation changes greatly and it has a higher measurement sensitivity to the temperature change in the optical fibre. After temperature compensation by Raman temperature measuring system, the winding strain curve is the same as the original strain, the correlation coefficient reaches 0.999 and the strain error is less than 50 με.

### 5.3. Winding Temperature Rise and Deformation Test

**Figure 12:**
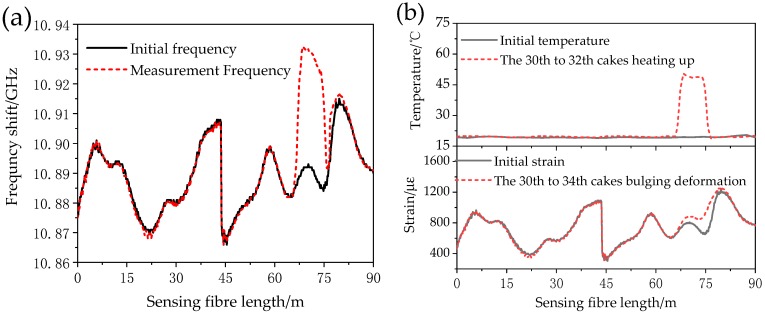
(**a**) The frequency shift curve of BOTDR and (**b**) the temperature and strain curves.

When the transformer encounters a short-circuit failure, the wire will experience sudden temperature changes due to the thermal effect of the short-circuit current while the winding is deformed due to the action of the short-circuiting electromotive forces. Therefore, the voltage of the resistance wires on the wires of the No. 30–32 cakes increases so as to increase the wire temperature to 40 °C and radial bulging deformation is set on the wire between two the adjacent stays of the No. 30–34 cakes. Figure 12a shows the frequency shift measurements before and after temperature compensation in the BOTDR system. The comparisons between the ROTDR measured temperature curve and compensated stress measuring curve and the original curves are shown in Figure 12b.

As shown in Figure 12, the frequency shift curve as measured by the BOTDR system is affected by temperature and strain. After a temperature curve is obtained from the ROTDR system measurements, the real strain curve is found. As shown in Table 4, the winding deformation, as measured by the BOTDR system, is greater than that of the temperature rise, which is consistent with the actual test settings. However, since the spatial resolution of the BOTDR system is set to be 2 m, the scope of measured sudden strain change is larger than the actual setting. Besides, since the measured optical fibre strain is the average strain within the spatial resolution, the measured optical fibre strain is much lower than the actual wire strain. In the next step, the relationship between system spatial resolution settings and measurement location accuracy will be studied so as to locate positions undergoing deformation more accurately.

## 6. Conclusions

A distributed optical fibre detection method for the temperature and strain of transformer winding based on Brillouin-Raman joint measuring was presented, an optical fibre composite transformer winding was designed and fabricated, and the temperature rise and deformation measurement tests were conducted. The following conclusions may be drawn:

- The wire grooving slightly increased the maximum inter-turn field strength, but was not enough to affect the inter-turn insulation strength of the wire; the embedded optical fibre was able to transfer the wire strain and temperature, and the change in strain, as measured by BOTDR, reflected the change of state of the winding.
- The optical fibre is subject to tensile and compressive forces during wire winding and a certain pre-stress developed after the wire was wound. The optical fibre shall be laid at the pressure withstanding side as much as possible to avoid the bending of the optical fibre.
- Brillouin-Raman joint measuring was able to distinguish the optical fibre temperature and strain information, and monitor (in real-time) the winding temperature and strain distribution, and locate hot-spots and deformation positions. At the same time, the strong insulation properties of the optical fibre determined its potential for the on-line monitoring of transformer state and could overcome the shortcomings in traditional detection methods.
- In future research, the correlation between the change of optical fibre strain curve and the winding wire deformation and type, and pattern recognition will be studied so as to provide more accurate state information for engineers responsible for transformer maintenance.

## Figures and Tables

**Table 1:** The dielectric constants of the material.

Material	Transformer Oil	Insulation Paper	Insulation Paint	Optical Fibre Core	Optical Fibre Coating
Relative dielectric constant	2.2	3.6	3.0	3.5	2.9

**Table 2:** The parameter settings.

ROTDR		BOTDR	
Temperature range/°C	−190~700	Strain range/%	±1.5
Accuracy/°C	±1	Pulse width/ns	20
Resolution/°C	≤0.5	Frequency sweep step/MHz	5
Response time/s	2~10	Calculation times	2^13^
Measuring distance/km	2	Measuring distance/km	1
Sample interval/m	0.4~0.8	Sample interval/m	0.2

**Table 3:** The comparison of results.

Test Results	First Measurement	Second Measurement
Temperature rise position/cake	10–12	30–32
Actual position/m	22.5–29.5	67.5–74.5
Measurement position/m	21–31.5	65.6–76.0
Mean optical fibre temperature/°C	39.2	59.6
Mean thermocouple temperature/°C	40.1	61.2

**Table 4:** The result comparison.

Test Results	Temperature Measurement	Strain Measurement
Temperature rise position/cake	30–32	-
Deformation position/cake	-	30–34
Actual position/m	67.5–74.5	67.5–79
Measuring position/m	65.8–76.5	65–82.2
Mean optical fibre temperature/°C	48.3	
Mean thermocouple temperature°C	50.2	
